# Relationship between mucosal TNF-α expression and Th1, Th17, Th22 and Treg responses in *Helicobacter pylori* infection

**DOI:** 10.1186/s13568-022-01456-0

**Published:** 2022-09-03

**Authors:** Ghorbanali Rahimian, Milad Shahini Shams Abadi, Yousef Mirzaei, Ali Hussein Mer, Reza Ahmadi, Fatemeh Azadegan-Dehkordi

**Affiliations:** 1 Department of Internal Medicine, Shahrekord University of Medical Sciences, Shahrekord, Iran; 2 Cellular and Molecular Research Center, Basic Health Sciences Institute, Shahrekord University of Medical Sciences, Shahrekord, Iran; 3 Department of Medical Biochemical Analysis, Cihan University-Erbil, Kurdistan Region, Iraq; 4 Scientific Research Center, Soran University, Soran, Kurdistan Region Iraq; 5 Clinical Biochemistry Research Center, Basic Health Sciences Institute, Shahrekord University of Medical Sciences, Shahrekord, Iran

**Keywords:** *Helicobacter pylori*, TNF-α, T helper, Peptic ulcer disease, Gastritis, Virulence factors

## Abstract

*Helicobacter pylori* (*H. pylori*)-induced gastric inflammation in the gastric mucosa and significantly increases the risk of developing gastritis and peptic ulcer disease (PUD). The objective of this research is to determine the role of tumor necrosis factor-α (TNF-α) expression in the gastric mucosa of patients with *H. pylori*-associated gastritis and PUD compared to uninfected patients, and we determined the relation between TNF-α expression and Th1/Th17/Th22, and Treg cells. Fifty-five patients with *H. pylori*-associated gastritis, 47 patients with *H. pylori*-associated PUD, and 48 uninfected patients were in this research. Antrum biopsy was used to detect *H. pylori,* virulence factors and histopathological assessments. Expression of TNF-α in the infected group was significantly higher than the uninfected group. Also, *cagA*/*oipA*-positive infected patients induce significantly more TNF-α expression than do *cagA*/*oipA*-negative infected patients. Expression of TNF-α was significantly increased in the PUD group than the gastritis group. Notably, TNF-α expression had a significant positive correlation with the frequency of Th1/Th17/Th22 lymphocytes in the PUD group. These findings indicate the importance of increasing TNF-α with Th1, Th17, Th22 responses increase as an important risk factor for PUD in context of *H. pylori* infection.

## Key points

- Expression of TNF-α was significantly increased in the PUD group than the gastritis group.
- Notably, TNF-α expression had a significant positive correlation with the frequency of Th1/Th17/Th22 lymphocytes in the PUD group.
- These findings indicate the importance of increasing TNF-α with Th1, Th17, Th22 responses increase as an important risk factor for PUD in context of *H. pylori* infection.

## Introduction

*Helicobacter pylori* (*H. pylori*) is a bacterial infection that causes stomach inflammation leading to a diversity of upper gastrointestinal disorderliness such as peptic ulcer disease (PUD), gastritis, and gastric cancer (GC) and mucosa-associated lymphoid tissue (MALT) lymphoma (Lu et al. 2012). This infection is related with histological gastritis and significant penetration of immune cell such as neutrophils, monocytes and lymphocytes (T and B cells) into the human gastric mucosa, which contribute toward maintaining as well as increasing the gastric inflammation (Azadegan-Dehkordi et al. 2020; Nahid-Samiei et al. 2020; Sanaei et al. 2021a). Activation and recruitment of immune cells site of infection is associated with the inflammatory process and the related cytokine production that can lead to gastric inflammation and subsequently causes gastric mucosal damage (Bagheri et al. 2015b; Sanaei et al. 2021b). The disorder is the outcome of the complex interaction among the host genetic factors, local innate and adaptive immune responses, *H. pylori* virulence factors, and environmental conditions (e.g. high salt intake, malnutrition, vitamin and antioxidants deficiency and smoking) (Shirzad et al. 2015; Yuzhalin 2011). Recent studies have shown that inflammatory and anti-inflammatory patterns in the gastric tissue samples are related with bacterial virulence factors (Ferreira et al. 2016; Rahimian et al. 2014). Clinical outcome after infection with *H. pylori* has been proposed to be associated with virulence factors of *H. pylori*, including the outer inflammatory protein a (OipA) and the cytotoxin-associated gene (*cagA*) (Bagheri et al. 2016a; El Khadir et al. 2018; Farzi et al. 2018). The CagA protein has been demonstrated to be involved in the up-regulation of inflammatory cytokines and higher grades of gastric inflammation that can lead to the extension of pathological conditions such as PUD and GC (Yamaoka et al. 2002).

OipA protein has been confirmed to be involved in the induction of a pro-inflammatory response such as IL-8, which is related with more severe neutrophil infiltration and high density of *H. pylori* (Javdan et al. 2019; Yamaoka et al. 2000). Tumor necrosis factor-α (TNF-α) is a multifunctional proinflammatory cytokine that regulates the immune inflammatory reaction (Thalmaier et al. 2002). TNF-α is produced primarily by macrophages, neutrophils, dendritic cells and T cells. TNF-α promotes inflammation by triggering the localized accumulation of immune cells and mediators of inflammation (Waters et al. 2013). In addition, TNF-α acts an important role in the mobility of inflammatory processes and tissue destruction such as autoimmune diseases (Redlich et al. 2002). The T helper (Th) cells that recognize as CD4 + cells, a type of T cell that have a major function in the production of various cytokines. Th1 cells, a Th cell subpopulation can produce a set of cytokines; cause the infiltration of pro-inflammatory M1 macrophages into the site of inflammation (Farrar et al. 2002). Th17 lineage has gained wide acceptance as a distinct subset of Th cell subpopulation (Noack and Miossec 2014). Th17 cells stimulate the epithelial and stromal cells to release proinflammatory cytokines and chemokines which recruit macrophages, lymphocytes, and neutrophils to the infection site (Bagheri et al. 2015a). Moreover, Th22, a novel Th cell subpopulation can trigger the secretion of TNF-α and IL-22 (Eyerich et al. 2009; Shohan et al. 2020). The regulatory T (Treg) lymphocytes are a subpopulation of Th cell that act to suppress immune response, maintain tolerance and prevention of uncontrolled inflammation (Bagheri et al. 2016c). Treg lymphocytes usually repress the increase of antigen-stimulated naive T cells (Bagheri et al. 2016c). Regarding the significance of Th1, Th17, Th22, and Treg responses and TNF-α cytokine in the context of inflammation and the probable roles in pathogenesis of *H. pylori*-infection, this research was done to study the role of TNF-α expression in the gastric mucosa of *H. pylori*-positive patients with PUD and gastritis compared to uninfected patients, and we determined the relation between TNF-α expression and the numeral of Treg, Th1, Th17, and Th22 cells in *H. pylori*-positive patients with PUD.

## Materials and methods

### Patients and sampling

The study population consisted of 48 uninfected patients with non-ulcer dysplasia (29 females, 19 males; age, 50.96 ± 19.77 years; range 30–65), 47 *H. pylori*-positive patients with PUD (20 females, 27 males; age, 50.16 ± 15.3 years; range 33–64) and 55 *H. pylori*-positive patients with gastritis (29 females, 26 males; age, 50.18 ± 15.02 years; range 29–67). This research was approved by the Shahrekord University of Medical Sciences Human Research Ethics Committee (Number IR.SKUMS.REC.1396.129), and prior to participation, obtaining informed consent was done from each volunteer. The rapid urease test, polymerase chain reaction (PCR), and histological examination of the biopsies taken from the body confirmed the *H. pylori* infection. So, if all four tests were positive, the patients classified as *H. pylori*-infected. 4 biopsies were gathered and used for the histological examination, rapid urease test, cytokine RNA analysis, detection of *H. pylori*, and assessment of the bacterial virulence factors.

### Immunohistochemistry and histological examination

For immunohistochemical examination, 4-µm serial sections were made and spread on poly-l-lysine coated slides. Paraffin sections were dried in a 70 °C oven for 8–12 h, deparaffinized in xylene for 3 times, 10 min each and hydrated using a series of alcohols (100%, 100%, 80% and 70%), 10 min each. Antigen retrieval was performed routinely by immersing the sections in citrate buffer (10 mM Sodium Citrate, 0.05% Tween 20, pH = 6.0) in a pressure cooker by autoclaving for 20 min. The sections were then incubated with protein block (ab93697, Abcam, Cambridge, UK) for 1 h to block nonspecific background staining. Subsequently, rabbit monoclonal anti-T-bet/Tbx21 antibody (ab150440, Abcam, Cambridge, UK) at a 1:400 dilution, rabbit anti-human RORγt antibody (ab80690, Abcam, Cambridge, UK) at a 1:350 dilution, rabbit anti-human IL-22 antibody (ab106773, Abcam, Cambridge, UK) at a 1:400 dilution and rabbit anti-human FOXP3 antibody (ab99963, Abcam, Cambridge, UK) at a 1:350 dilution were applied for detection of Th1, Th17, Th22 and Treg cells, respectively. In the following, the sections that were latter incubated overnight in a humidified chamber at 4 °C. On the second day, endogenous peroxidase activity was blocked with 3% H_2_O_2_ in TBS for 15 min. Afterwards, Biotinylated goat anti-rabbit and mouse IgG (ab93697, Abcam, Cambridge, UK) was applied and the sections were incubated for 1 h at room temperature. Then, applying Streptavidin Peroxidase Plus, the sections were incubated for 10 min at room temperature. Afterwards Applying DAB (ab94665, Abcam, Cambridge, UK) to tissue, the sections were incubated for 10 min. Sections were counterstained for 1 min with Meyer’s hematoxylin and then mounted. Human hodgkin's lymphoma tissue was used as a positive control. Additional sections were processed without primary antibody as a negative control. The number of Th1, Th17, Th22 and Treg cells was calculated by counting positive lymphocytes throughout the entire area of tissue section at 10 high power fields (Bagheri et al. 2018, 2017, 2019; Sanaii et al. 2019). Results were expressed as the mean value and interquartile range of all tested patients in each group.

For the histopathological estimate, a biopsy sample was taken of gastric antral mucosa. The fixed samples in 10 percent buffered formalin for 24 h, paraffin-embedded, tissue sections (4-μm) stained sequentially with hematoxylin and eosin for grading and assessing gastritis severity and stained with modified Giemsa because *H. pylori* can be visualized by light microscopy. Colonized *H. pylori* in gastric epithelial have been Classified on a scale of four points including mild, moderate, and severe colonization. We described an ulcer as a mucosal break > 3 mm in the exudate covered duodenum or stomach. The slides were tested blinded using a pathologist to the specifications of *H. pylori* infection. Polymorph nuclear cell infiltration and mononuclear cells infiltrating was classified and scored according to Sydney’s system: normal = 0, mild = 1, moderate = 2 and severe = 3 (Dixon et al. 1996).

### PCR amplification

Genomic DNA was extracted by Bio spin Kit (Bio Flux, Japan) for the polymerase chain reaction (PCR). Sequences of primers and PCR reaction conditions we have before explained by Salimzadeh et al. (2015).

### Quantitative analysis of the TNF-α expression using quantitative polymerase chain reaction (qPCR)

Total RNA from the biopsy samples was extracted using a TRIzol® Plus RNA Purification Kit according to the supplier’s instructions. Complementary DNA (cDNA) was synthesized using reverse transcriptase (RT) using the First Strand cDNA Synthesis Kit (Fermentas Life Sciences, cat- K1622). For cDNA synthesis 2.5 microgram of pure RNA was used as template. Using the TaqMan RT-PCR system, amplification of TNF-α and β-actin cDNA was performed in a Rotorgene 3000 (Corbett Research). The real time-PCR reactions were performed in a total volume of 20 μl containing 5.75 μl of nuclease-free H2O, 3 μl of synthesized cDNA solution, 10 μl of 2x Rotor-Gene Probe PCR Master Mix (Qiagen, Germany), 0.5 μl of each primer (10 pM) and 0.25 μl of the TaqMan probe (10 pM). Negative controls for real time-PCR amplification were prepared by omitting the cDNA sample from the reaction mixture. Thermal cycling was initiated with a first denaturation step at 95 °C for 10 min and followed by 45 cycles of 95 °C for 15 s and 60 °C for 60 s. Oligonucleotide sequences of primer and probe used in this research are as follows: TNF-α (Forward, 5-TCTTCTCGAACCCCGAGTGA-3; Reverse, 5-CCTCTGATGGCACCACCAG-3; probe, 5-FAM-TAGCCCATGTTGTAGCAAACCCTCAAGCT-TAMRA-3). The relative expression was computed based on the expression ratio of a target gene (TNF-α) versus a reference gene (β-actin). The 2^−ΔΔCt^method was the determining approach of the relative quantification of the ratio of cytokine to β-actin (Bagheri et al. 2016b).

### Data analysis

All data provided are expressed as mean ± SEM. Checking normality tests done using the Shapiro-Wilk. Statistical analysis was done by t-test for independent samples and one-way ANOVA test, chi-squared (χ^2^) test, Pearson’s correlation and only values with equal *P-*values or less than 0.05 were determined as significant.

## Results

### Significant relationship of TNF-α mRNA expression with *H. pylori* infection

**Fig. 1:**
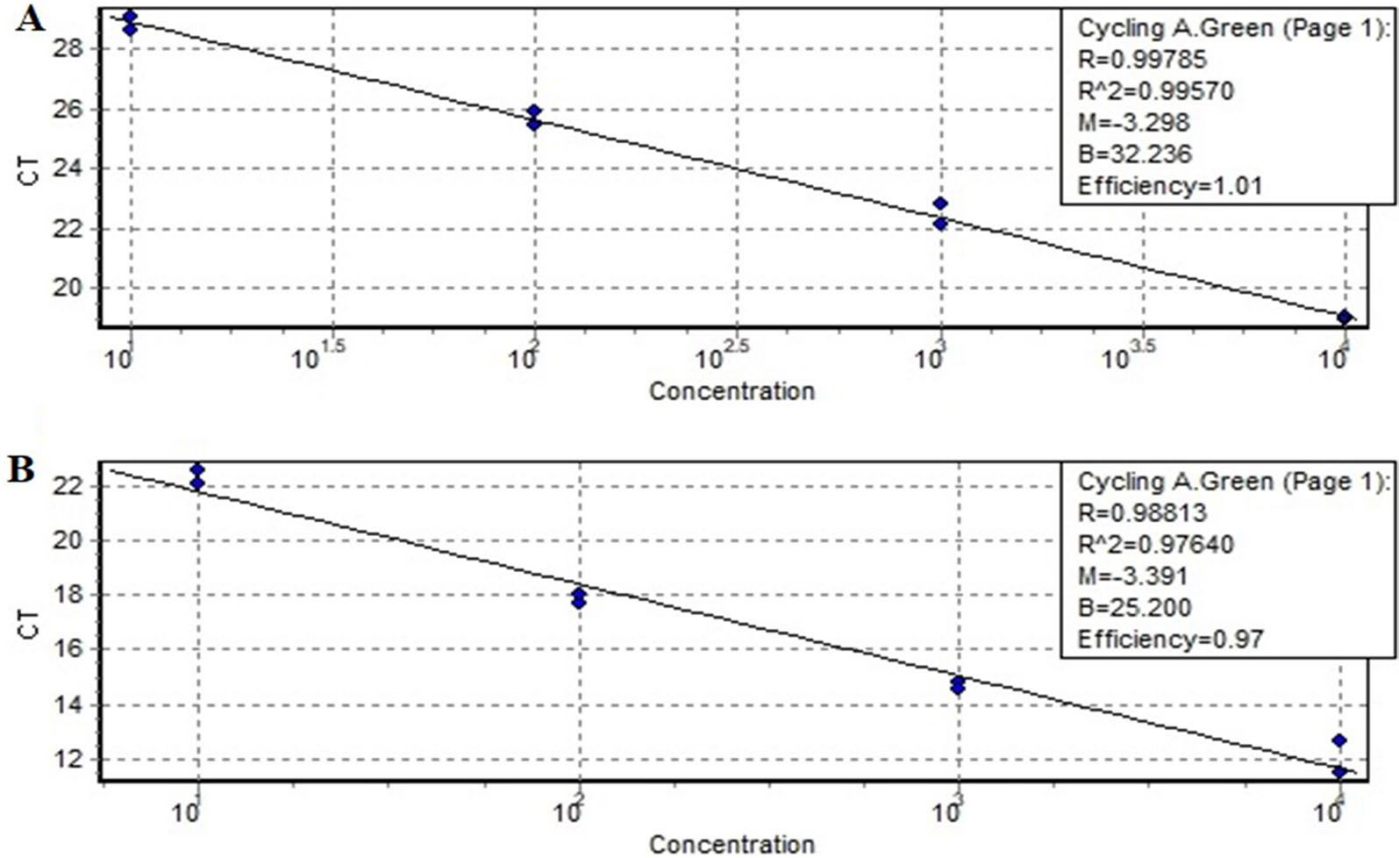
PCR efficiency of qPCR primers drawn by standard curve assay. **A** The efficiency of β-actin primer was 101%. **B** The efficiency of TNF-α primer was 97%. PCR efficiency of TaqMan probe and primers is stated with 100% ± 5%

**Fig. 2:**
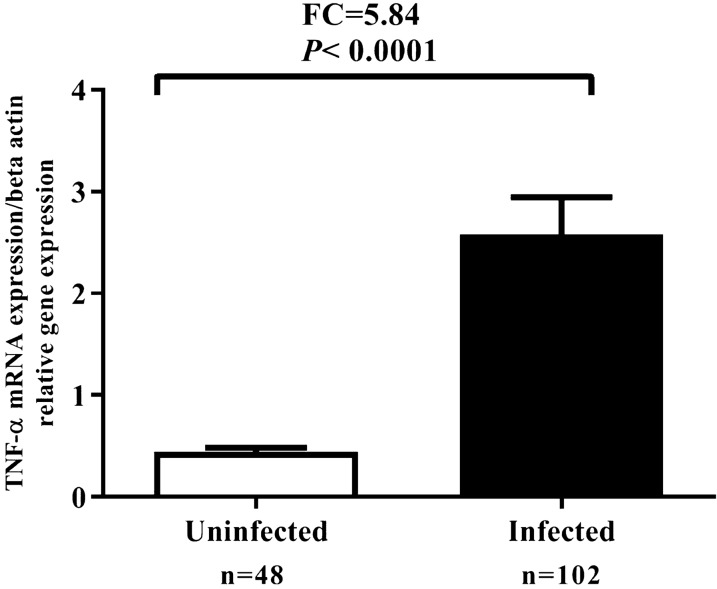
Comparison of TNF-α mRNA expression in patients with or without *H. pylori*-infection. TNF-α expression was significantly increased in *H. pylori-*infected individuals in comparison with uninfected patients. *P* < 0 .05 was considered statistically significant by independent samples t-test (N = number and FC = fold change)

To create a standard curve, we need to create a serial dilution series. We used 1:10, 1:100, 1:1000, and 1:10,000 serial dilution to create a standard curve. PCR efficiency of TaqMan probe and primers is stated with 100% ± 5%. PCR efficiency of qPCR primers for β-actin and TNF-α was analyzed by a standard curve assay and is shown in Fig. 1. The TNF-α mRNA expression was significantly higher in *H. pylori*-infected gastric tissue in comparison with uninfected gastric tissue (Fig. 2). TNF-α fold change in infected group compared to non-infected group was 5.84-fold.

### Significant relationship of TNF-α mRNA expression with *H. pylori* virulence factor *cagA* and *oipA*

**Fig. 3:**
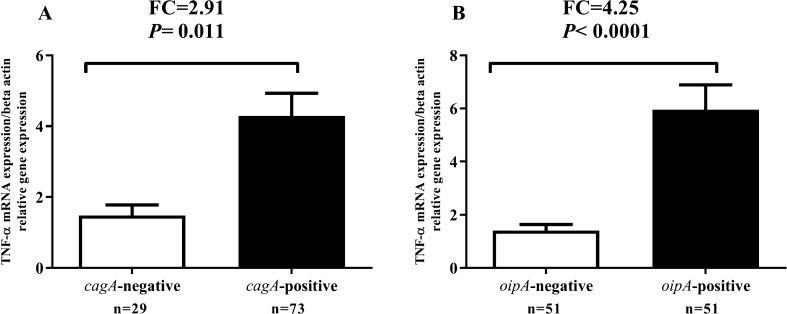
Comparison of TNF-α mRNA expression in patients infected with or without the *cagA* and *oipA* virulence factors. TNF-α expression was significantly increased in patients infected with *cagA*-positive in comparison with patients infected with *cagA*-negative. Also, TNF-α expression was significantly increased in patients infected with *oipA*-positive in comparison with patients infected with *oipA*-negative*. P* < 0 .05 was considered statistically significant by independent-samples t-test (N = number and FC = fold change)

We investigated whether *H. pylori* virulence factors *cagA* and *oipA*, which are the main virulence factors, were related to the changes observed in TNF-α mRNA expression. Interestingly, the TNF-α mRNA expression was significantly higher in patients infected with *cagA*-positive (*cagA*^+^) and *oipA*-positive *(oipA*^+^) in comparison with in patients infected with *cagA*-negative (*cagA*^-^) and *oipA*-negative *(oipA*^*-*^) patients (Fig. 3A-B). TNF-α fold change in *cagA*^+^ and *oipA*^+^ groups compared to *cagA*^*-*^ and *oipA*^*-*^ groups was 2.91-fold and 4.25-fold, respectively.

### Relation of acute inflammatory score and *H. pylori* virulence factor *cagA* and *oipA*

The association between acute inflammatory score and *H. pylori* virulence factor *cagA* and *oipA* is described in Table 1. *H. pylori* virulence factors *cagA*, *oipA* have a significant association to acute inflammatory score. Therefore, *H. pylori* virulence factor *cagA* and *oipA* enhancement acute inflammatory score.

**Table 1:** Association between score of acute inflammatory with the *oipA* and *cagA* virulence factors in infected patients

Virulence factors	Acute inflammatory score [n (%)]				Total [n (%)]	*P* values^a^
	Negative	Mild	Moderate	Severe		
*oipA*-Positive	7 (31.8)	26 (46.4)	12 (75)	6 (75)	51 (50)	0.004
*oipA*-Negative	15 (68.2)	30 (53.6)	4 (25)	2 (25)	51 (50)	
*cagA*-Positive	13 (59.1)	39 (69.6)	13 (81.3)	8 (100)	73 (71.6)	0.019
*cagA*-Negative	9 (40.9)	17 (30.4)	3 (18.7)	0 (0.0)	29 (28.4)	

^a^Fisher’s exact test

### Status of *oipA* and *cagA* genes of *H. pylori* from infected patients with PUD and gastritis

In this research, we have checked the association among the presence of *oipA* and *cagA* genes of *H. pylori* and the different gastro duodenal diseases. Also, *oipA* and *cagA* genes were not shown remarkable differences among *H. pylori-*positive subjects with gastritis and PUD (*P*> 0.05) (Table 2).

**Table 2:** Status of *oipA* and *cagA* gene of *H. pylori* in PUD and gastritis infected individuals

Virulence factors	PUD [n (%)]	Gastritis [n (%)]	Total [n (%)]	*P* values ^a^
*oipA*-Positive	26 (50.3)	25 (45.5)	51 (50)	0.321
*oipA*-Negative	21 (44.7)	30 (54.5)	51 (50)	
*cagA*-Positive	34 (72.3)	39 (70.9)	73 (71.6)	0.873
*cagA*-Negative	13 (27.7)	16 (29.1)	29 (28.4)	

^a^Chi-Square test

### Comparison of TNF-α mRNA expression at *H. pylori*-positive subjects with PUD and gastritis

**Fig. 4:**
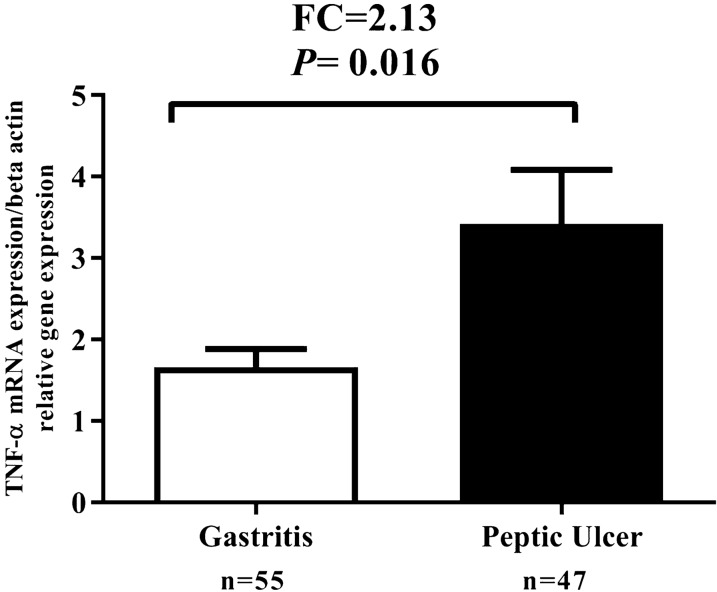
Comparison of TNF-α mRNA expression in PUD and gastritis *H. pylori-*infected individuals. TNF-α mRNA expression was significantly increased in PUD *H. pylori-*infected individuals than gastritis *H. pylori-*infected individuals. *P* < 0 .05 was considered statistically significant by independent-samples t-test (N = number and FC = fold change)

Meaningful expression of TNF-α mRNA increased at *H. pylori*-positive patients with PUD than *H. pylori*-positive patients with gastritis (Fig. 4). TNF-α fold change in *H. pylori*-positive patients with PUD compared to *H. pylori*-positive patients with gastritis was 2.13-fold.

### Significant relationship of TNF-α mRNA expression with scoring acute inflammatory, chronic inflammation score and the grade of density of *H. pylori* in infected subjects

**Fig. 5:**
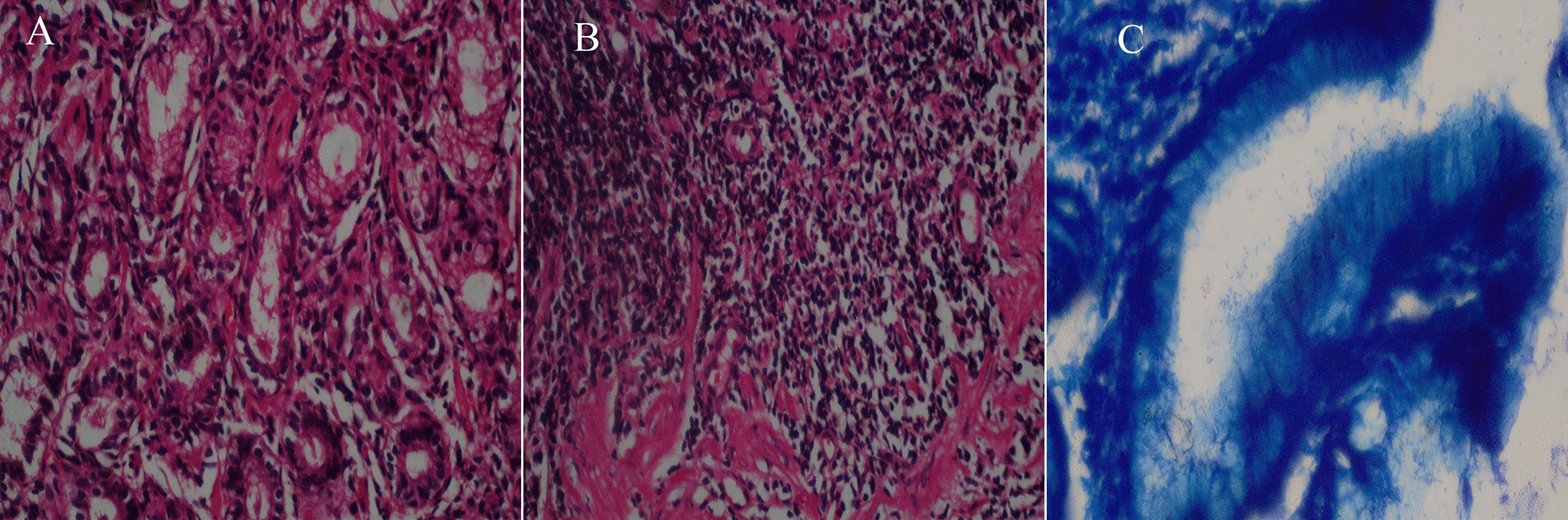
Histological examination. **A** Gastric pits infiltrated by neutrophils in a case of *H. pylori*-positive. **B** Severe chronic gastritis with chronic inflammatory cells presents in the superficial lamina propria in excess of normal. **C** *H. pylori* organisms are present in the mucous layer on the gastric mucosal surface (400x)

**Fig. 6:**
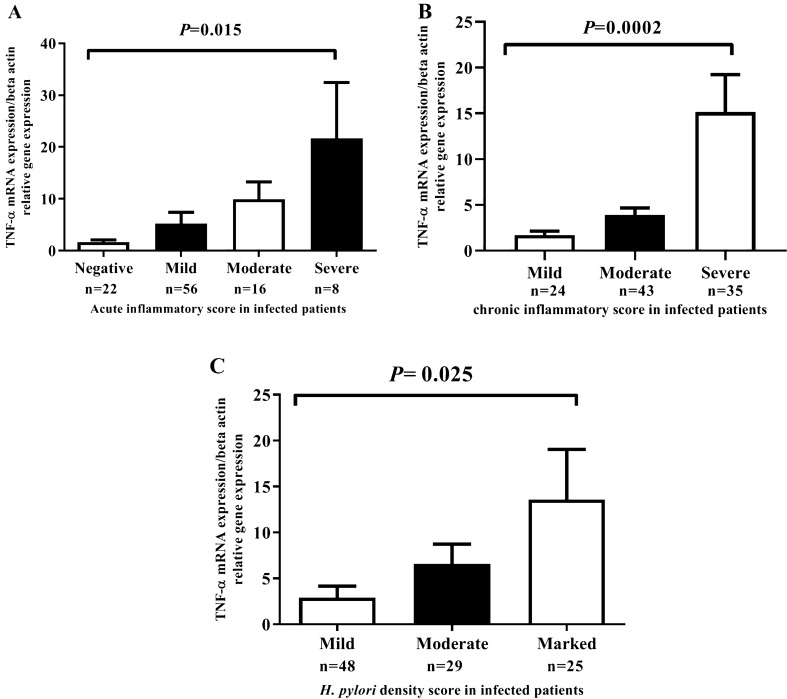
The correlation between TNF-α mRNA expression and acute inflammatory score, chronic inflammatory score and the grad of the density of *H. pylori* infection in patients. Increase in acute inflammatory score, the score of chronic inflammation and the *H. pylori* density grade is significantly related to increase TNF-α mRNA expression in infected patients. *P* < 0 .05 was determine statistically significant by one-way ANOVA (N = number)

The grade of polymorph nuclear cell and mononuclear cells, and *H. pylori* density in the gastric mucosa of were determined in two consecutive sections (Fig. 5). Also, an enhancement in chronic, acute inflammation score and the grade of infection density is significantly related to increased TNF-α mRNA expression in *H. pylori*-infected subjects (Fig. 6A: *P* = 0.015, Fig. 6B: *P*= 0.0002, and Fig. 6C: *P* = 0.025, respectively).

### Correlation between mucosal TNF-α mRNA expression and frequency of Th1, Th17, Th22 lymphocytes and Treg lymphocytes in *H. pylori*-positive patients with PUD

**Fig. 7:**
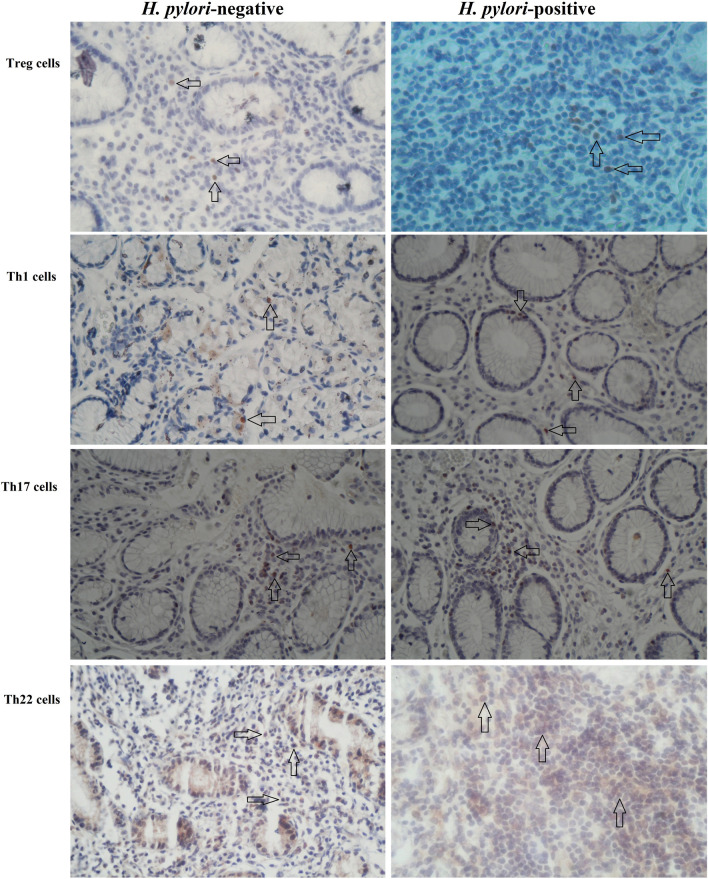
Immunohistochemistry of Treg, Th1, Th17, and Th22 cells in *H. pylori*-negative subjects and *H. pylori*-positive subjects (original magnification, 400 ×). Immunohistochemistry of Tregs (row 1), Th1 (row 2), Th17 (row 3), and Th22 (row 4) in gastric mucosa from column 1) *H. pylori*-negative subjects and column 2) *H. pylori*-positive subjects. IL-22 staining (brown) were found on the cytoplasm of T lymphocytes, and Foxp3 (Treg), T-bet (Th1) and RORγ (Th17) staining (brown) was located in the nucleus of T lymphocytes. The arrows show the target lymphocytes in the gastric tissue

**Fig. 8:**
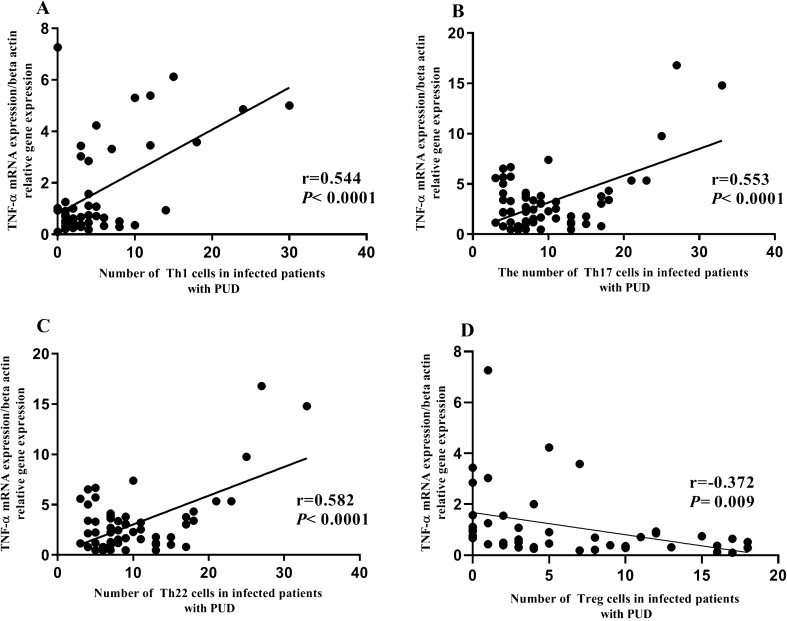
The association between TNF-α mRNA expression and the numeral of Th1, Th17, Th22, and Treg lymphocytes in PUD *H. pylori*-positive patients. There was a significant positive association between expression of TNF-α and frequency of Th1, Th17, Th22 lymphocytes, whilst a significant negative association was shown between TNF-α expression and Treg lymphocytes in PUD *H. pylori*-positive patients. The data were analyzed using Spearman's rank correlation

The number of Treg, Th1, Th17, and Th22 cells in the gastric mucosa of gastroduodenal diseases were determined in two consecutive sections (Fig. 7). The mucosal TNF-α mRNA expression had a significant positive correlation with the frequency of Th1, Th17, Th22 lymphocytes in *H. pylori*-positive patients with PUD (Fig. 8A: r = 0.554, *P*< 0.0001, Fig. 8B: r = 0.553, *P*< 0.0001 and Fig. 8C: r = 0.582, *P*< 0.0001, respectively). But, the mucosal TNF-α mRNA expression had a significant negative correlation with the frequency of Treg lymphocytes in *H. pylori*-positive patients with PUD (Fig. 8E: r = − 0.372, *P*= 0.009).

## Discussion

The host immune responses to *H. pylori* in infected patients strongly stimulate the inflammation of the gastric mucosa, which appeared to have played an important role in the various clinical outcomes including gastritis and PUD (Bagheri et al. 2018, 2019). It has been indicated that *H. pylori*-infection is one of the main risk factors for PUD that the mucous membrane is disordered and it causes gastric and duodenal inflammation (Rosenstock et al. 2003). *H. pylori* cannot be the main reason for PUD; however, *H. pylori*-induced gastric membrane inflammation may lead to various clinical outcomes (Bagheri et al. 2018, 2019).

Our outcomes indicated that TNF-α mRNA expression in *H. pylori*-infected patients was higher in comparison with that of *H. pylori* negative patients. These findings are in agreement with previous reports that the TNF-α expression has been demonstrated to increase in the gastric mucosa of *H. pylori*-infected patients (Collodel et al. 2010; Kumar Pachathundikandi et al. 2011; Shamsdin et al. 2017; Tourani et al. 2018). It has been shown that *H. pylori* infection on AGS and gastric epithelial cells induces nuclear factor kappa-light-chain-enhancer of activated B cells (NF-κB) activation (Maeda et al. 2000). NF-κB enters the nucleus in response to certain stimuli, including proinflammatory cytokines such as TNF-α and IL-8 (Keates et al. 1997; Maeda et al. 2000). Several studies have demonstrated that polymorphisms in the *TNF-α* genes result in high transcriptional promoter activity along with increased gastric inflammation, which are related with an increased risk of *H. pylori*- related GC and PUD developments (El-Omar et al. 2003; Sugimoto et al. 2007). The expression of the TNF-α in the infected patients with *-cagA*^*-*^ and *oipA*^+^ were significantly higher than the infected patients with *cagA*^*-*^ and *oipA*^-^negative. Also, the attendance of *oipA*, *cagA* virulence factors increments acute inflammatory score. Kumar Pachathundikandi *et al*. have shown that *H. pylori*-infection induced the transcription factor NF-κB stimulation and release of IL-8 and TNF-α in a cagPAI-dependent way in THP-1 monocytes, which was associated with enhanced TLR-2 and TLR-5 expressions (Kumar Pachathundikandi et al. 2011). Research in infected Mongolian gerbils with *H. pylori* showed that the mucosal pattern of inflammatory cytokines such as IL-1β, TNF-α, IL-17, and IL-18 in infected Mongolian gerbils with *oipA* mutant were significantly lower than infected with wild-type strains. Also, *H. pylori* virulence factors OipA determined the levels of the gastric inflammation-related inflammatory cytokine outcome in the progress of GC and PUD (Sugimoto et al. 2009). TNF-α mRNA expression in the infected patients with PUD was significantly higher than in the patients with gastritis. Also, an increase in acute inflammatory score, chronic inflammation score and the degree of *H. pylori* density is significantly associated with increased TNF-α mRNA expression in infected patients. Our results are in agreement with the results of the studies conducted on mouse models of the *H. pylori*-infection, which demonstrated that the TNF-α mRNA expression were significantly greater in the PUD group than in the non-ulcer group (Sugimoto et al. 2009). Also, recent study by Tourani et al. Showed that the serum TNF-α level in peptic ulcer patients was significantly higher than the control group (Tourani et al. 2018). In contrast to our detections, Shamsdin et al. demonstrated that and noted no significant change in TNF-α serum levels in the infected patients with PUD and gastritis; however, the TNF-α serum levels in combination with IL-6 were found to be significantly higher in the gastritis patients as compared to the PUD patients. In addition, they observed significantly higher levels of TNF-α in the active chronic and chronic gastritis patients as compared to the uninfected groups (Shamsdin et al. 2017).

The mucosal TNF-α mRNA expression had a significant positive correlation with the frequency of Th1, Th17, Th22 lymphocytes in the PUD group. Our previous studies in association with the pathogenesis of PUD showed that the frequency of Th1, Th17, Th22 lymphocytes in infected patients with PUD were significantly higher than infected patients with gastritis (Bagheri et al. 2018, 2019; Sanaii et al. 2019). Th1 cells cause the infiltration of pro-inflammatory M1 macrophages into the site of inflammation (Farrar et al. 2002). M1 macrophages are usually characterized by the secretion of high levels of pro-inflammatory cytokines such as TNF-α and IL-6 (Atri et al. 2018). Th17 lymphocytes stimulate epithelial cells and stromal cells to release IL-8, which is a chemo attractant for neutrophils, and Th17 lymphocytes also induces the production of TNF-α from macrophages and neutrophils cells at the site of infection (Bagheri et al. 2015a). Also, the pro-inflammatory cytokine TNF-α may promote Th22 differentiation in the site of inflammation (Sugita et al. 2013). The increased TNF-α mRNA expression may be able to reflect the degree of tissue damage affected by a peptic ulcer in comparison to gastritis patients. The mucosal TNF-α mRNA expression had a significant negative correlation with the frequency of Treg lymphocytes in the PUD group. Our previous study showed that the frequency of Treg and Breg lymphocytes remarkably increased in gastritis patients rather than PUD patients (Bagheri et al. 2017; Nahid-Samiei et al. 2020).

The findings suggest that increased levels of TNF-α mRNA in *H. pylori*-infected patients may act an axial role in PUD pathogenesis. The conclusions as well as highlighted the significance of TNF-α increment along with Th1, Th17, Th22 responses increase also a significant risk factor for PUD in the context of *H. pylori* infection.

## Data Availability

The data are available. All data generated or analyzed during this study are included in this study.

## Abbreviations

*H.** pylori*: *Helicobacter pylori*
PUD: Peptic ulcer disease
TNF-α: Tumor necrosis factor-α
GC: Gastric cancer
MALT: Mucosa-associated lymphoid tissue
oipA: Outer inflammatory protein a
cagA: Cytotoxin-associated gene
Th: T helper
Treg: Regulatory T

## References

[CR1] Atri C, Guerfali FZ, Laouini D (2018). Role of human macrophage polarization in inflammation during infectious diseases. Int J Mol Sci.

[CR2] Azadegan-Dehkordi F, Shirzad H, Ahmadi R, Bashash D, Abdollahpour-Alitappeh M, Luzza F, Larussa T, Nahid-Samiei M, Rahimian G, Shafigh MH, Bagheri N (2020). Increased indoleamine 2, 3-dioxygenase expression modulates Th1/Th17/Th22 and Treg pathway in humans with *Helicobacter **pylori*-Infected gastric mucosa. Hum Immunol.

[CR3] Bagheri N, Azadegan-Dehkordi F, Shirzad H, Rafieian-Kopaei M, Rahimian G, Razavi A (2015). The biological functions of IL-17 in different clinical expressions of *Helicobacter pylori*-infection. Microb Pathog.

[CR4] Bagheri N, Azadegan-Dehkordi F, Shirzad M, Zamanzad B, Rahimian G, Taghikhani A, Rafieian-Kopaei M, Shirzad H (2015). Mucosal interleukin-21 mRNA expression level is high in patients with *Helicobacter pylori* and is associated with the severity of gastritis. Cent Eur J Immunol.

[CR5] Bagheri N, Azadegan-Dehkordi F, Rafieian-Kopaei M, Rahimian G, Asadi-Samani M, Shirzad H (2016). Clinical relevance of *Helicobacter pylori* virulence factors in Iranian patients with gastrointestinal diseases. Microb Pathog.

[CR6] Bagheri N, Azadegan-Dehkordi F, Rahimian G, Hashemzadeh-Chaleshtori M, Rafieian-Kopaei M, Kheiri S, Gholipour A, Shirzad H (2016). Altered Th17 cytokine expression in *Helicobacter pylori* patients with TLR4 (D299G) polymorphism. Immunol Invest.

[CR7] Bagheri N, Azadegan-Dehkordi F, Rahimian G, Rafieian-Kopaei M, Shirzad H (2016). Role of regulatory T-cells in different clinical expressions of *Helicobacter pylori* infection. Arch Med Res.

[CR8] Bagheri N, Shirzad H, Elahi S, Azadegan-Dehkordi F, Rahimian G, Shafigh M, Rashidii R, Sarafnejad A, Rafieian-Kopaei M, Faridani R, Tahmasbi K, Kheiri S, Razavi A (2017). Downregulated regulatory T cell function is associated with increased peptic ulcer in *Helicobacter pylori*-infection. Microb Pathog.

[CR9] Bagheri N, Razavi A, Pourgheysari B, Azadegan-Dehkordi F, Rahimian G, Pirayesh A, Shafigh M, Rafieian-Kopaei M, Fereidani R, Tahmasbi K, Shirzad H (2018). Up-regulated Th17 cell function is associated with increased peptic ulcer disease in *Helicobacter pylori*-infection. Infect Genet Evol.

[CR10] Bagheri N, Shirzad H, Mirzaei Y, Nahid-Samiei M, Sanaei M, Rahimian G, Shafigh M, Zandi F, Tahmasbi K, Razavi A (2019). T-bet(+) cells polarization in patients infected with *Helicobacter pylori* Increase the risk of peptic ulcer development. Arch Med Res.

[CR11] Collodel G, Moretti E, Campagna MS, Capitani S, Lenzi C, Figura N (2010). Infection by CagA-positive *Helicobacter pylori* strains may contribute to alter the sperm quality of men with fertility disorders and increase the systemic levels of TNF-α. Dig Dis Sci.

[CR12] Dixon MF, Genta RM, Yardley JH, Correa P (1996). Classification and grading of gastritis. The updated Sydney System. International Workshop on the Histopathology of Gastritis, Houston 1994. Am J Surg Pathol.

[CR13] El Khadir M, Alaoui Boukhris S, Benajah DA, Ibrahimi SA, Chbani L, Bouguenouch L, El Rhazi K, El Abkari M, Nejjari C, Mahmoud M, Bennani B (2018). *Helicobacter pylori* CagA EPIYA-C motifs and gastric diseases in Moroccan patients. Infect Genet Evol J Mol Epidemiol Evol Genet Infect Dis.

[CR14] El-Omar EM, Rabkin CS, Gammon MD, Vaughan TL, Risch HA, Schoenberg JB, Stanford JL, Mayne ST, Goedert J, Blot WJ, Fraumeni JF, Chow WH (2003). Increased risk of noncardia gastric cancer associated with proinflammatory cytokine gene polymorphisms. Gastroenterology.

[CR15] Eyerich S, Eyerich K, Pennino D, Carbone T, Nasorri F, Pallotta S, Cianfarani F, Odorisio T, Traidl-Hoffmann C, Behrendt H, Durham SR, Schmidt-Weber CB, Cavani A (2009). Th22 cells represent a distinct human T cell subset involved in epidermal immunity and remodeling. J Clin Investig.

[CR16] Farrar JD, Asnagli H, Murphy KM (2002). T helper subset development: roles of instruction, selection, and transcription. J Clin Investig.

[CR17] Farzi N, Yadegar A, Aghdaei HA, Yamaoka Y, Zali MR (2018). Genetic diversity and functional analysis of oipA gene in association with other virulence factors among *Helicobacter pylori* isolates from Iranian patients with different gastric diseases. Infect Genet Evol.

[CR18] Ferreira RM, Pinto-Ribeiro I, Wen X, Marcos-Pinto R, Dinis-Ribeiro M, Carneiro F, Figueiredo C (2016). *Helicobacter pylori* cagA promoter region sequences influence CagA expression and interleukin 8 secretion. J Infect Dis.

[CR19] Javdan S, Narimani T, Abadi MSS, Gholipour A (2019). Agr typing of *Staphylococcus aureus* species isolated from clinical samples in training hospitals of Isfahan and Shahrekord. BMC Res Notes.

[CR20] Keates S, Hitti YS, Upton M, Kelly CP (1997). *Helicobacter pylori* infection activates NF-kappa B in gastric epithelial cells. Gastroenterology.

[CR21] Kumar Pachathundikandi S, Brandt S, Madassery J, Backert S (2011). Induction of TLR-2 and TLR-5 expression by *Helicobacter pylori* switches cagPAI-dependent signalling leading to the secretion of IL-8 and TNF-alpha. PLoS ONE.

[CR22] Lu Y, Giver CR, Sharma A, Li JM, Darlak KA, Owens LM, Roback JD, Galipeau J, Waller EK (2012). IFN-gamma and indoleamine 2,3-dioxygenase signaling between donor dendritic cells and T cells regulates graft versus host and graft versus leukemia activity. Blood.

[CR23] Maeda S, Yoshida H, Ogura K, Mitsuno Y, Hirata Y, Yamaji Y, Akanuma M, Shiratori Y, Omata M (2000). *H. pylori* activates NF-kappaB through a signaling pathway involving IkappaB kinases, NF-kappaB-inducing kinase, TRAF2, and TRAF6 in gastric cancer cells. Gastroenterology.

[CR24] Nahid-Samiei M, Rahimian G, Shafigh M, Taheri F, Karami-Hurestani M, Sanaei MJ, Heshmati M, Bagheri N (2020). Enhanced frequency of CD19(+)IL-10(+)B cells in human gastric mucosa infected by *Helicobacter pylori*. Am J Med Sci.

[CR25] Noack M, Miossec P (2014). Th17 and regulatory T cell balance in autoimmune and inflammatory diseases. Autoimmun Rev.

[CR26] Rahimian G, Sanei MH, Shirzad H, Azadegan-Dehkordi F, Taghikhani A, Salimzadeh L, Hashemzadeh-Chaleshtori M, Rafieian-Kopaei M, Bagheri N (2014). Virulence factors of *Helicobacter pylori* vacA increase markedly gastric mucosal TGF-beta1 mRNA expression in gastritis patients. Microb Pathog.

[CR27] Redlich K, Hayer S, Maier A, Dunstan CR, Tohidast-Akrad M, Lang S, Turk B, Pietschmann P, Woloszczuk W, Haralambous S, Kollias G, Steiner G, Smolen JS, Schett G (2002). Tumor necrosis factor alpha-mediated joint destruction is inhibited by targeting osteoclasts with osteoprotegerin. Arthritis Rheum.

[CR28] Rosenstock S, Jørgensen T, Bonnevie O, Andersen L (2003). Risk factors for peptic ulcer disease: a population based prospective cohort study comprising 2416 Danish adults. Gut.

[CR29] Salimzadeh L, Bagheri N, Zamanzad B, Azadegan-Dehkordi F, Rahimian G, Hashemzadeh-Chaleshtori M, Rafieian-Kopaei M, Sanei MH, Shirzad H (2015). Frequency of virulence factors in *Helicobacter pylori*-infected patients with gastritis. Microb Pathog.

[CR30] Sanaei MJ, Nahid-Samiei M, Abadi MSS, Arjmand MH, Ferns GA, Bashash D, Rahimian G, Bagheri N (2021). New insights into regulatory B cells biology in viral, bacterial, and parasitic infections. Infect Genet Evol.

[CR31] Sanaei MJ, Shirzad H, Soltani A, Abdollahpour-Alitappeh M, Shafigh MH, Rahimian G, Mirzaei Y, Bagheri N (2021). Up-regulated CCL18, CCL28 and CXCL13 expression is associated with the risk of gastritis and peptic ulcer disease in *Helicobacter pylori* infection. Am J Med Sci.

[CR32] Sanaii A, Shirzad H, Haghighian M, Rahimian G, Soltani A, Shafigh M, Tahmasbi K, Bagheri N (2019). Role of Th22 cells in *Helicobacter pylori*-related gastritis and peptic ulcer diseases. Mol Biol Rep.

[CR33] Shamsdin SA, Alborzi A, Rasouli M, Ghaderi A, Lankrani KB, Dehghani SM, Pouladfar GR (2017). The importance of TH22 and TC22 cells in the pathogenesis of *Helicobacter pylori*-associated gastric diseases. Helicobacter.

[CR34] Shirzad H, Bagheri N, Azadegan-Dehkordi F, Zamanzad B, Izadpanah E, Abdi M, Ramazani G, Sanei MH, Ayoubian H, Ahmadi A, Jamalzehi S, Aslani P, Zandi F (2015). New insight to IL-23/IL-17 axis in Iranian infected adult patients with gastritis: effects of genes polymorphisms on expression of cytokines. Acta Gastroenterol Belg.

[CR35] Shohan M, Dehghani R, Khodadadi A, Dehnavi S, Ahmadi R, Joudaki N, Houshmandfar S, Shamshiri M, Shojapourian S, Bagheri N (2020). Interleukin-22 and intestinal homeostasis: protective or destructive?. IUBMB Life.

[CR36] Sugimoto M, Furuta T, Shirai N, Nakamura A, Xiao F, Kajimura M, Sugimura H, Hishida A (2007). Different effects of polymorphisms of tumor necrosis factor-alpha and interleukin-1 beta on development of peptic ulcer and gastric cancer. J Gastroenterol Hepatol.

[CR37] Sugimoto M, Ohno T, Graham DY, Yamaoka Y (2009). Gastric mucosal interleukin-17 and -18 mRNA expression in *Helicobacter pylori*-induced Mongolian gerbils. Cancer Sci.

[CR38] Sugita S, Kawazoe Y, Imai A, Kawaguchi T, Horie S, Keino H, Takahashi M, Mochizuki M (2013). Role of IL-22- and TNF-alpha-producing Th22 cells in uveitis patients with Behcet's disease. J Immunol.

[CR39] Thalmaier U, Lehn N, Pfeffer K, Stolte M, Vieth M, Schneider-Brachert W (2002). Role of tumor necrosis factor alpha in *Helicobacter pylori* gastritis in tumor necrosis factor receptor 1-deficient mice. Infect Immun.

[CR40] Tourani M, Habibzadeh M, Karkhah A, Shokri-Shirvani J, Barari L, Nouri HR (2018). Association of TNF-α but not IL-1β levels with the presence of *Helicobacter pylori* infection increased the risk of peptic ulcer development. Cytokine.

[CR41] Waters JP, Pober JS, Bradley JR (2013). Tumour necrosis factor in infectious disease. J Pathol.

[CR42] Yamaoka Y, Kwon DH, Graham DY (2000). A M(r) 34,000 proinflammatory outer membrane protein (oipA) of *Helicobacter pylori*. Proc Natl Acad Sci USA.

[CR43] Yamaoka Y, Souchek J, Odenbreit S, Haas R, Arnqvist A, Borén T, Kodama T, Osato MS, Gutierrez O, Kim JG (2002). Discrimination between cases of duodenal ulcer and gastritis on the basis of putative virulence factors of *Helicobacter pylori*. J Clin Microbiol.

[CR44] Yuzhalin A (2011). The role of interleukin DNA polymorphisms in gastric cancer. Hum Immunol.

